# Supplementary material for: Major genetic discontinuity and novel toxigenic species in Clostridioides difficile taxonomy
Source: eLife. 2021 Jun 11;10:e64325. doi: 10.7554/eLife.64325 (PMC8241443; doi:10.7554/eLife.64325)
Supplement: Transparent reporting form [file elife-64325-transrepform.pdf]

eLIFE
Westbrook Centre, Milton Road
Cambridge CB4 1YG
UK
P 01223 855340
W elifesciences.org
T @elife

eLife's transparent reporting form

We encourage authors to provide detailed information *within their submission* to facilitate the interpretation and replication of experiments. Authors can upload supporting documentation to indicate the use of appropriate reporting guidelines for health-related research (see EQUATOR Network), life science research (see the BioSharing Information Resource), or the ARRIVE guidelines for reporting work involving animal research. Where applicable, authors should refer to any relevant reporting standards documents in this form.

If you have any questions, please consult our Journal Policies and/or contact us: editorial@elifesciences.org.

Sample-size estimation

- You should state whether an appropriate sample size was computed when the study was being designed
- You should state the statistical method of sample size computation and any required assumptions
- If no explicit power analysis was used, you should describe how you decided what sample (replicate) size (number) to use

Please outline where this information can be found within the submission (e.g., sections or figure legends), or explain why this information doesn't apply to your submission:

Sample size estimation did not apply to this study. We included all available *C. difficile* genomes in the public database as described in the following sections. This resulted in the largest possible sample size. For Bayesian analysis, the parameters used to achieve suitable sample sizes for each estimated variable were given in the methods section.

Replicates

- You should report how often each experiment was performed
- You should include a definition of biological versus technical replication
- The data obtained should be provided and sufficient information should be provided to indicate the number of independent biological and/or technical replicates
- If you encountered any outliers, you should describe how these were handled
- Criteria for exclusion/inclusion of data should be clearly stated
- High-throughput sequence data should be uploaded before submission, with a private link for reviewers provided (these are available from both GEO and ArrayExpress)

Please outline where this information can be found within the submission (e.g., sections or figure legends), or explain why this information doesn't apply to your submission:

eLife Sciences Publications, Ltd is a limited liability non-profit non-stock corporation incorporated in the State of Delaware, USA, with company number 5030732, and is registered in the UK with company number FC030576 and branch number BR015634 at the address Westbrook Centre, Milton Road Cambridge CB4 1YG, UK | March 2019

Replicates: Key genomic approaches used in this study included Bayesian evolutionary analyses, whole genome ANI analyses, and pangenome analyses. All three approaches used two independent algorithms and data for both is presented in the paper and detailed in the methods. Bayesian evolutionary analyses were performed using two independent algorithms (BEAST/BactDating). Whole genome ANI analyses were performed using two independent algorithms (FastANI, and pyani). Pangenome analyses were performed using two independent algorithms (Panaroo, and Roary).

High-throughput sequence data: Regarding the submission question - Did your work use any previously published datasets (e.g., DNA sequence data, clinical trial data, field data)? We retrieved the entire collection of *C. difficile* genomes (taxid ID 1496) held at the NCBI Sequence Read Archive [https://www.ncbi.nlm.nih.gov/sra/]. The raw dataset (as of 1st January 2020) comprised 12,621 genomes. These genomes comprise hundreds, maybe thousands of publications. The individual accession numbers for all genomes analysed in this study are provided in the Supplementary Data at http://doi.org/10.6084/m9.figshare.12471461.

Criteria for exclusion/inclusion of data should be clearly stated: This information is included within the methods and summarized here. Inclusion: All *Clostridioides difficile* genome sequences (taxid: 1496) available in the NCBI Sequence Read Archive as of 1st January 2020. https://www.ncbi.nlm.nih.gov/sra/. Exclusion: Only Illumina PE data was included in the study. Genomes were screened for contamination using Kraken2 against mini Kraken database. All genomes with < 85% reads identified as *Clostridioides difficile* are considered to be contaminated and excluded from the study.

Outliers: There were MLST outliers which could not be confidently assigned to an evolutionary clade. These were not analysed in the study. This is detailed in the results section.

Statistical reporting

- Statistical analysis methods should be described and justified
- Raw data should be presented in figures whenever informative to do so (typically when N per group is less than 10)
- For each experiment, you should identify the statistical tests used, exact values of N, definitions of center, methods of multiple test correction, and dispersion and precision measures (e.g., mean, median, SD, SEM, confidence intervals; and, for the major substantive results, a measure of effect size (e.g., Pearson's r, Cohen's d)
- Report exact p-values wherever possible alongside the summary statistics and 95% confidence intervals. These should be reported for all key questions and not only when the p-value is less than 0.05.

Please outline where this information can be found within the submission (e.g., sections or figure legends), or explain why this information doesn't apply to your submission:

> Statistical analysis methods are described and justified in the methods and within the relevant results sections e.g. CI values for Bayesian analyses (Fig 4), and p-values for pangenome analyses (Fig 6).

(For large datasets, or papers with a very large number of statistical tests, you may upload a single table file with tests, Ns, etc., with reference to sections in the manuscript.)

Group allocation

- Indicate how samples were allocated into experimental groups (in the case of clinical studies, please specify allocation to treatment method); if randomization was used, please also state if restricted randomization was applied
- Indicate if masking was used during group allocation, data collection and/or data analysis

Please outline where this information can be found within the submission (e.g., sections or figure legends), or explain why this information doesn't apply to your submission:

> Genomes were allocated into evolutionary clades based on MLST (https://pubmlst.org/). This information is found in the results (Fig 1) and Supplementary Data which is hosted at Figshare http://doi.org/10.6084/m9.figshare.12471461.

Additional data files ("source data")

- We encourage you to upload relevant additional data files, such as numerical data that are represented as a graph in a figure, or as a summary table
- Where provided, these should be in the most useful format, and they can be uploaded as "Source data" files linked to a main figure or table
- Include model definition files including the full list of parameters used
- Include code used for data analysis (e.g., R, MatLab)
- Avoid stating that data files are "available upon request"

Please indicate the figures or tables for which source data files have been provided:

> Supplementary Data files on figshare include: [1] Full MLST data for all 12000+ *C. difficile* genomes (Fig 1); [2] Whole-genome ANI analyses (Table 1, Fig 3, Fig 5); [3] Tree files for phylogenetic analyses (Fig 2, Fig 4); [4] Pangenome data (Fig 6); [5] Pan-GWAS data (Table 2); and [6] Comparative genomic analysis of virulence gene architecture (Fig 7).

eLife Sciences Publications, Ltd is a limited liability non-profit non-stock corporation incorporated in the State of Delaware, USA, with company number 5030732, and is registered in the UK with company number FC030576 and branch number BR015634 at the address Westbrook Centre, Milton Road Cambridge CB4 1YG, UK | March 2019
